# Enhancing both the Intensities and Resolution of ^19^F NMR Spectra of PFAS through Band-Selective Homonuclear Decoupling

**DOI:** 10.1021/acs.analchem.5c05889

**Published:** 2026-01-27

**Authors:** Markus Rotzinger, Viktoria Müller, Armin Macher, Jörg Feldmann, Klaus Zangger

**Affiliations:** † Institute of Chemistry/Organic and Bioorganic Chemistry, 27267 University of Graz, Heinrichstraße 28, 8010 Graz, Austria; ‡ Institute of Chemistry/Analytical Chemistry, 27267 University of Graz, Universitätsplatz 1, 8010 Graz, Austria; § The James Hutton Institute, Craigiebuckler, Aberdeen AB15 8QH, United Kingdom

## Abstract

A method for acquiring fluorine nuclear magnetic resonance (^19^F NMR) spectra of perfluoroalkyl and polyfluoroalkyl substances (PFASs) is described. The experiment uses band-selective homonuclear decoupling of the CF_3_ spectral region (around −82.4 ppm) and results in spectra showing both enhanced resolution and enhanced signal intensities for perfluorinated compounds. PFAS mixtures, which show highly overlapped multiplet signals in conventional ^19^F NMR spectra, are reduced to individual separated singlets, enabling not only their identification but also quantification, which does not depend on the chemical nature of each compound.

## Graphical abstract

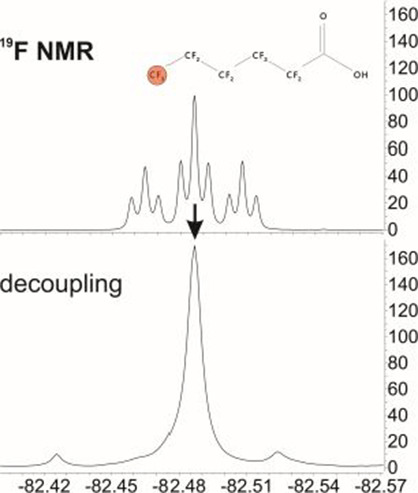

## 1 Introduction

Per- and polyfluoroalkyl substances (PFASs) are a diverse class of substances defined by the presence of perfluorinated carbon atoms.^1^ PFASs, which were developed more than 70 years ago, exhibit several favorable properties, which made them indispensable in the manufacturing of various items such as nonstick coatings of pans, food-packaging, and firefighting foams.^2^ However, several of the compounds have been identified to bioaccumulate at alarming levels and are potentially toxic.^3–5^ The exceptional strength of the carbon–fluorine bond makes PFASs very resistant to degradation, which is the reason that they are commonly referred to as “forever chemicals”. Through leaching from consumer products or pollution from PFAS production plants, PFAS contaminations in soil, water, air, and food are found in many different sites. PFAS exposure has been linked to a variety of health impacts. It is associated with increased risk of various cancers (kidney, testicular, and prostate cancers), liver damage and immune system dysfunction.^6^ Very recently, several studies indicated a link between PFAS exposure and the risk to develop dementia.^7,8^ Due to these negative health effects, the detection and quantification of PFASs have become a major focus in analytical chemistry.

A recent study evaluating PFAS standards showed that conventional LC/GC-MS spectrometry failed to detect the analyte for about 20% of the substances in a high-throughput screening setup.^9^ Due to the vast variations in physicochemical properties of PFASs, no individual LC- or GC-MS technique can be able to identify and quantify all possible PFASs at once. NMR spectroscopy could serve as an alternative approach to PFAS detection without relying on ionization or chromatography. Only recently, efforts have been made to establish ^19^F NMR as a complementary technique for the analysis of PFAS samples. A fluorine NMR spectroscopic analysis typically does not depend on matrix interference, and no sample cleanup is needed. Additionally, the spectra can be analyzed quantitatively, even for unknown PFASs, without the need for calibration of each compound. The applications have been limited by the relatively low sensitivity of ^19^F NMR and the limited resolution, especially when the CF_3_ region is analyzed in PFAS mixtures.^10,11^ Therefore, the fluorine NMR analysis of PFASs has been often restricted to reference compounds or samples at relatively high concentrations.^12–15^

^19^F spectra of PFAS compounds show distinct signals for the CF_3_ groups around −82.4 ppm, which can be used to identify compounds irrespective of the headgroup.^10^ However, these signals exhibit complex spin multiplicities due to the extensive scalar coupling networks present in perfluorinated compounds.^16^ The induced signal splitting imposes a limit in signal separation and hampers the qualitative and especially quantitative analysis of mixtures due to spectral overlap.

Here, we introduce a method that uses band-selective homonuclear decoupling (BASH) to collapse the split signals of the CF_3_ groups into a single sharp signal concomitant with an enhancement in signal-to-noise ratios. Therefore, this approach enhances both the sensitivity and resolution of ^19^F NMR spectra in a selected spectral region. The method employed is a ^19^F-variant of the BASH decoupling method, which has previously been applied to the recording of ultrahigh resolution proton-decoupled peptide NOESY spectra.^17–20^ BASH decoupling is a band-selective variant of real-time pure-shift NMR techniques,^21–23^ which achieve homonuclear decoupling through repeated refocusing of scalar coupling during acquisition.^24–26^ This approach allows the acquisition of the highest resolution ^19^F spectra of PFASs while also boasting improved signal intensities due to the collapse of the multiplicity into a singlet peak. The CF_3_ region represents an optimal target for PFAS mixture analysis because CF_3_ groups occur in many PFASs and this spectral region is free of overlapping CF_2_ resonances. At the same time, it still provides enough chemical shift dispersion to resolve individual compounds within a mixture.^11^

## 2 Materials and Methods

### 2.1 Sample Preparation

Stock solutions of the solid perfluoroalkyl and polyfluoroalkyl substances (PFAS) standards were prepared in methanol. Solutions of perfluorocarboxylic acids (PFCAs) were neutralized with equimolar amounts of sodium hydroxide to prevent esterification during sample preparation. Mixed PFAS samples were prepared from stock solutions and transferred into 5 mm NMR tubes. A methanol-d_4_ solution of chromium­(III) acetylacetonate was added to achieve a final concentration of 4 mg mL^–1^ to shorten longitudinal relaxation times (T_1_) from 1.9 to 0.6 s and enable reduced relaxation delays between scans while keeping T_2_ largely unaffected. The total sample volume was adjusted to 550 μL with an additional methanol-d_4_. Methanol-d_4_ was purchased from Eurisotop (Saarbrücken, Germany).

### 2.2 NMR Measurements

All NMR spectra were acquired at 298 K on a Bruker Avance Neo 500 MHz spectrometer running topspin 4.1.3 equipped with a 5 mm SEF ^19^F probe at a ^19^F frequency of 470.54 MHz. Hard-pulse ^19^F excitation parameters were optimized iteratively to 5 μs from a 360° pulse, and 90° soft pulses were calculated from the determined hard-pulse values. Spectra of PFAS mixtures at 1.5 mg/L (Figure 3) were collected with 16,384 transients and an 8192-point complex time domain. A relaxation delay of 1 s was applied to qualitative spectra. The transmitter offset was set to −82.3 ppm, and a band-selective pulse with an excitation bandwidth of 2500 Hz was used. Chemical shift referencing was achieved using an internal CFCl_3_ standard. Quantitative spectra (Figure 4) were acquired under identical conditions using 1024 transients and a relaxation delay of 10 s to ensure full spin relaxation between scans. The spectra were processed using standard Bruker processing with an exponential window function.

Sample concentrations for the quantifications (Figure 4) were approximately 10 mg L^–1^, with perfluoropropionic acid (PFPrA) added as an internal standard at 1 mg L^–1^. Quantitative results were obtained by comparing the fitted integrals of the analyte and internal standard resonances, normalized by molecular mass. Each measurement was recorded in triplicate under identical conditions in direct succession. Detailed acquisition parameters for the individual experiments are provided in Table S1.

## 3 Results and Discussion

### 3.1 NMR Method Development

The use of a ^19^F-variant of a real-time BASH decoupling (Figure 2) allows for a high resolution and high signal-to-noise experiment, permitting excitation bandwidths, which cover the entire CF_3_ region (5000 Hz), while leaving CF_2_ resonances unaffected. This ensures full homonuclear decoupling of the spectral region of interest.

Figure 1a–c illustrates the gain in resolution achievable via the removal of scalar coupling. The expansion of the CF_3_ region, depicted in 1b, shows a triplet of triplets due to the scalar couplings reaching up to ^4^J within the perfluorinated aliphatic chain. Homonuclear band-selective decoupling allows for the removal of the complex multiplicities and yields a pure-shift spectrum of the selected spectral region without the loss of signal to noise associated with related broadband decoupling methods. The band-selective pulses can be tuned easily to excite the CF_3_ region of several PFASs, as it is well-separated from the CF_2_ resonances in the chain, thus posing an ideal target for this approach. The refocusing of scalar coupling during acquisition is realized by interrupting the acquisition with a refocusing element consisting of a 180° hard pulse and a 180° band-selective pulse, each flanked by gradient pulses (1d) for artifact suppression. The interval at which this refocusing element is cycled, termed chunking time, depends on the scalar coupling constants and is in the same order of magnitude as in the related ^1^H variant of the experiment. For PFASs, we found an optimum chunking time of 21.7 ms.

**Figure 1:**
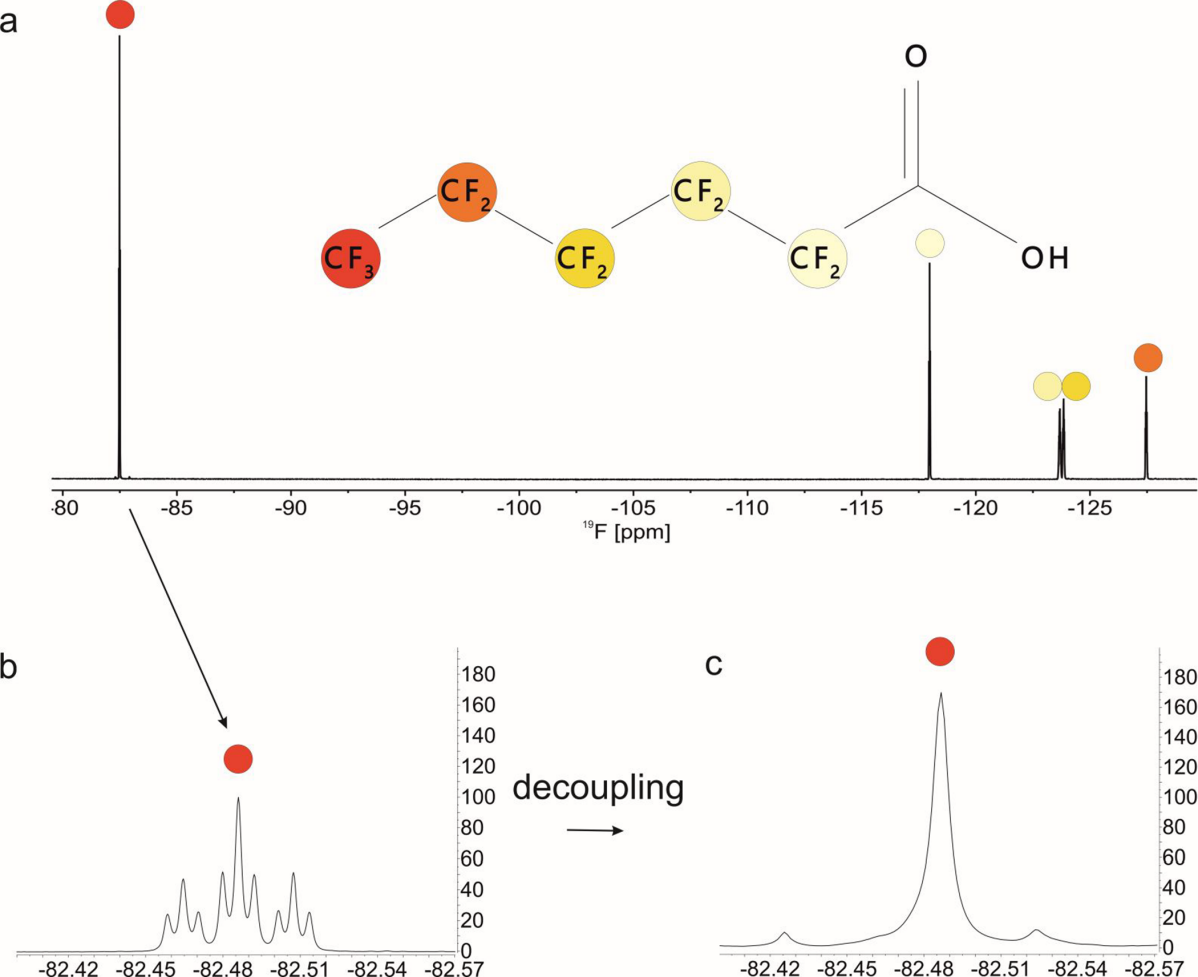
(a) ^19^F spectrum of perfluorohexanoic acid (PFHxA) in methanol-d_4_ at a concentration of 1000 mg/L. (b) Expansion of the CF_3_ spectral region taken from the same spectrum, showing a triplet of triplets multiplicity. (c) Expansion of the same CF_3_ spectral region from a band-selective decoupled ^19^F spectrum showing the collapse of the multiplet into a singlet.

**Figure 2:**
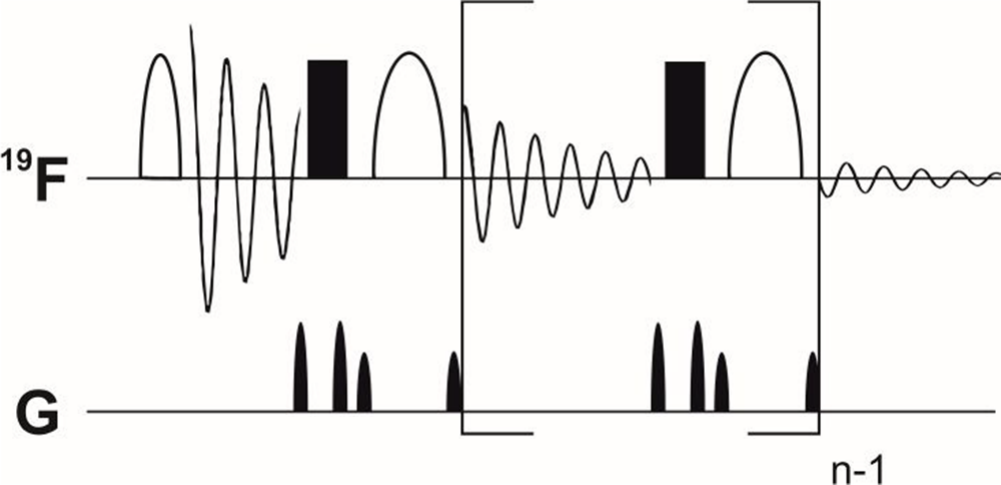
Pulse sequence of the employed band-selective decoupling sequence. Narrow half-ellipses indicate a 90° selective pulse, and wide half-ellipses a 180° selective pulse. Filled rectangles represent 180° hard pulses. Gradient pulses of different strengths are indicated on the lower line. Parentheses indicate the part of the pulse program that is repeated to ensure continuous refocusing of scalar coupling during acquisition.

To illustrate the increase in signal intensities as well as resolution, we recorded spectra of a mixture of 7 PFAS:6 perfluorocarboxylic acids of varying chain lengths (c4, c6, c7, c8, c9, c10) and one perfluorosulfonic acid (s6) dissolved in methanol at two different concentrations of 100 mg/L (Figure 3a) and 1 mg/L (Figure 3d). The expansion of the CF_3_ area (Figure 3b) shows limited spectral resolution due to overlaps of the triplet of triplet signals of the individual PFAS. The overlap of scalar coupled signals does not permit an unambiguous interpretation of the spectra. In contrast, the band-selectively decoupled spectrum of the CF_3_ region allows for a much clearer assignment (Figure 3f), since each visible peak belongs to an individual PFAS. The expanded regions, depicted in Figure 3e,f are baseline noise-matched to better visualize the effect of band-selective decoupling on sensitivity, which amounts to an increase in the signal-to-noise ratio of about 2-fold. Focusing on a limited spectral region also provides an additional advantage in the increase in signal per time, as acquisition times could be shortened to 0.81 s while still maintaining an adequate number of data points at a spectral width of 10 ppm. A key factor in optimizing the generated signal over time is the addition of 4 mg/mL of Cr­[Acac]_3_ as a paramagnetic relaxation additive, which allows for significantly faster pulsing.^11^

**Figure 3:**
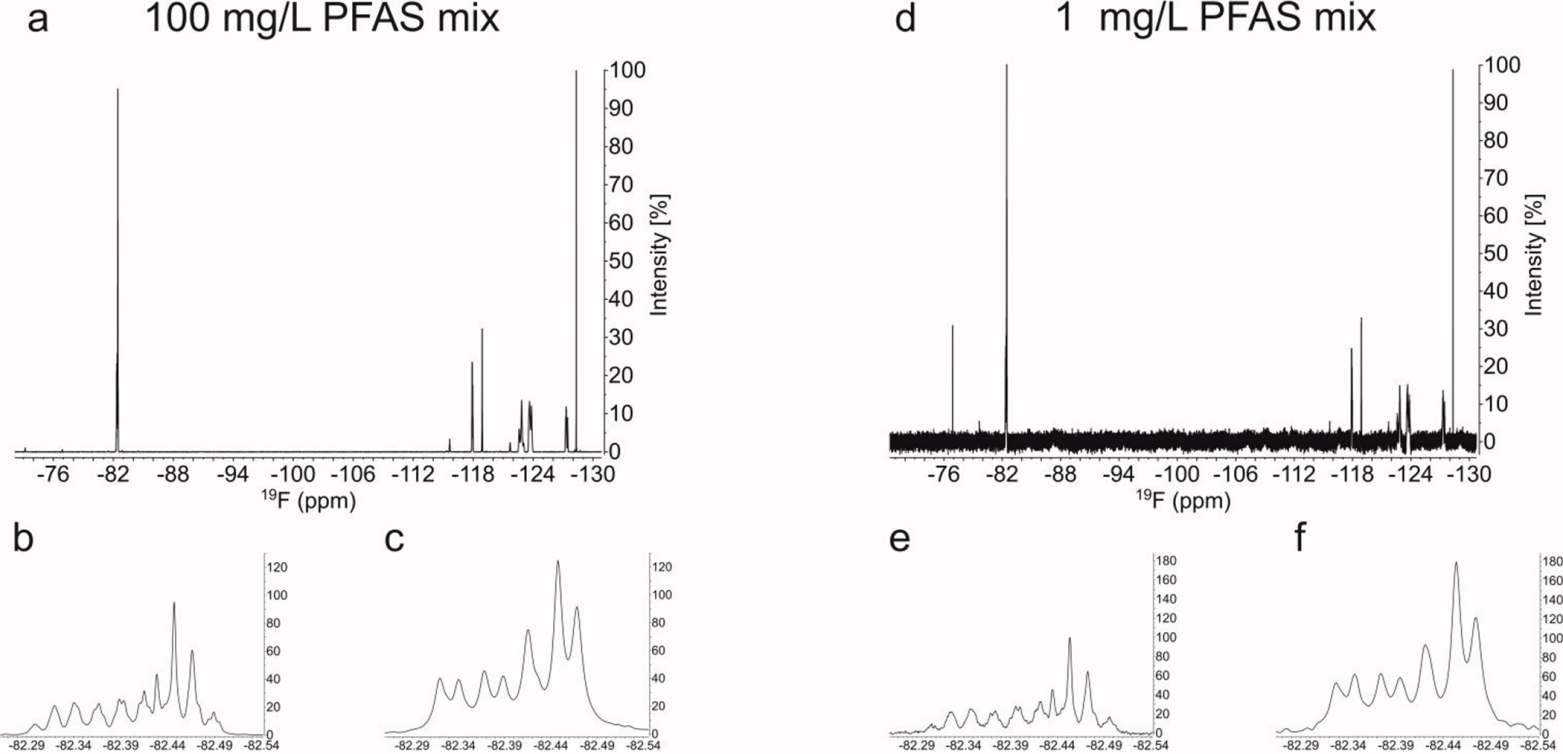
Comparison of ^19^F spectra of a mixture of 6 perfluorocarboxylic acids of varying chain lengths (c4, c6, c7, c8, c9, c10) and one perfluorosulfonic acid (s6) dissolved in methanol-d_4_: (a) spectrum recorded at 100 mg/L, (b) expansion of the CF_3_ region of (a), (c) expansion of the CF_3_ region of a band-selectively decoupled spectrum of the same sample, (d) ^19^F spectrum of PFHxA in methanol-d_4_ recorded at 1 mg/L, (e) expansion of the CF_3_ region of the spectrum in (d) with a matched signal-to-noise ratio, and (f) expansion of the CF_3_ region of a band-selectively decoupled spectrum of the same sample.

### 3.2 Increased Resolution Allows for Assignment of Resonances in a 7-Component Mixture

To demonstrate the ability to resolve a complex mixture of related PFASs, we applied this method to a mixture of 7-PFAS compounds recorded at concentrations of 1.5 mg/L. We were able to identify all of the individual compounds in the mixture via chemical shift matching to samples of the individual pure compounds within chemical shifts of 0.005–0.01 ppm (Figure 4). Sample identification from a mixture proved feasible, as using CFCl_3_ as an internal standard turned out to be sufficiently accurate for unambiguous matching. This was enabled by homonuclear decoupling of the signals, which removed any of the ambiguities in the evaluation of the data where otherwise overlaps due to multiplicity are present. However, great caution needs to be taken to ensure fully identical sample conditions, like solvent and temperature as even small changes may result in ambiguity for the resonance matching. Another factor to be considered is the effect of higher concentration of analytes in the sample on the chemical shift. If there is a significant amount of total acidic PFASs present in the sample, chemical shifts vary in comparison to the low-concentration sample due to pH effects caused by the acidity of the compounds. For the purpose of resolution limit testing, the methodology was applied to a significantly more complex sample in the 30-component HPLC recovery standard PFAC30PAR, which is given in Figure S3. In this case, even though the resolution is increased significantly, a lack of signal dispersion prevents an unambiguous matching of all resonances.

**Figure 4:**
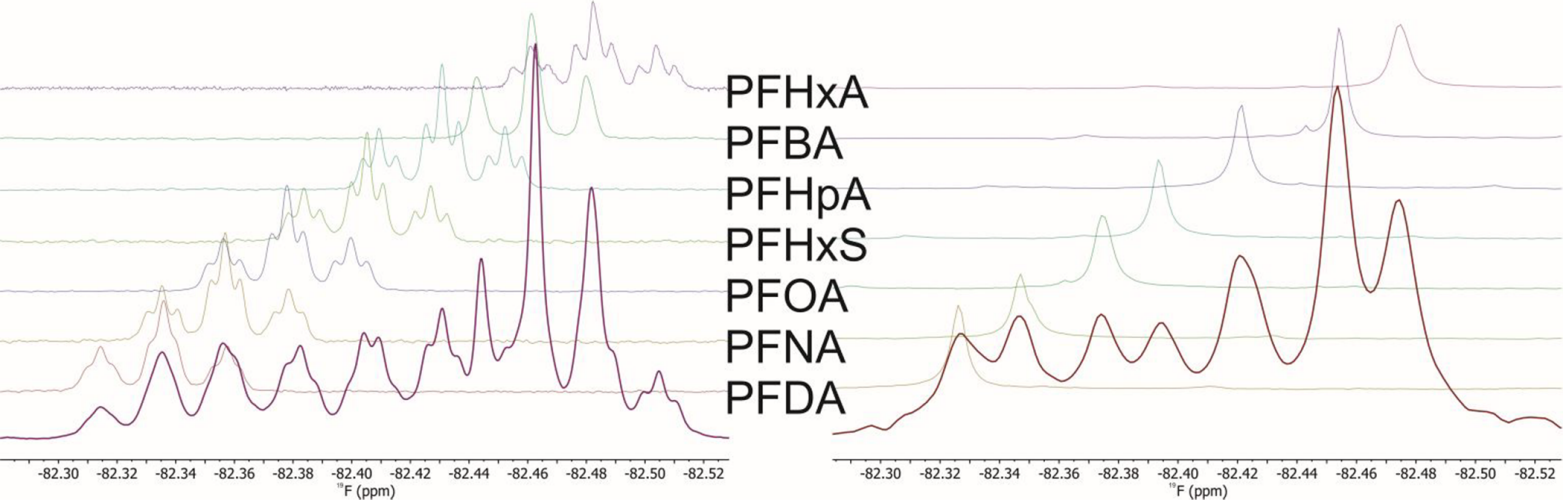
Spectra of a 7-PFAS mixture containing perfluorocarboxylic acids of varying chain lengths (c4, c6, c7, c8, c9, and c10) and one perfluorosulfonic acid (s6) at a concentration of 330 mg/L for the individual spectra and 1.5 mg/L for the PFAS mix. The respective coupled and decoupled stacked spectra of the individual compounds were recorded on the same samples. The spectra were referenced to CFCl_3_ via internal referencing.

### 3.3 Resolution and Sensitivity Are Sufficient for Quantification in a 3-Compound Mixture

To compare results obtained with the band-selectively decoupled ^19^F NMR with LC-MS data, a quantitative study on a 3-compound mixture was conducted. The concentrations could be reliably determined to be within 90% of the expected values. The line fitting algorithm in MestreNova allowed for a facile extraction of accurate integrals even in overlapped peaks in the mixtures of perfluorocarboxylic acids of similar, medium to long chain lengths (Figure 5a). In addition to PFOA and PFNA, 10:2 fluorotelomeric alcohol (10:2 FTOH) was simultaneously quantified in the case of the NMR sample, yielding similar concentrations of 6.9 mg/L, demonstrating the tolerance of the method when chemically diverse compounds are present and CF_3_ signals overlap partially.

**Figure 5:**
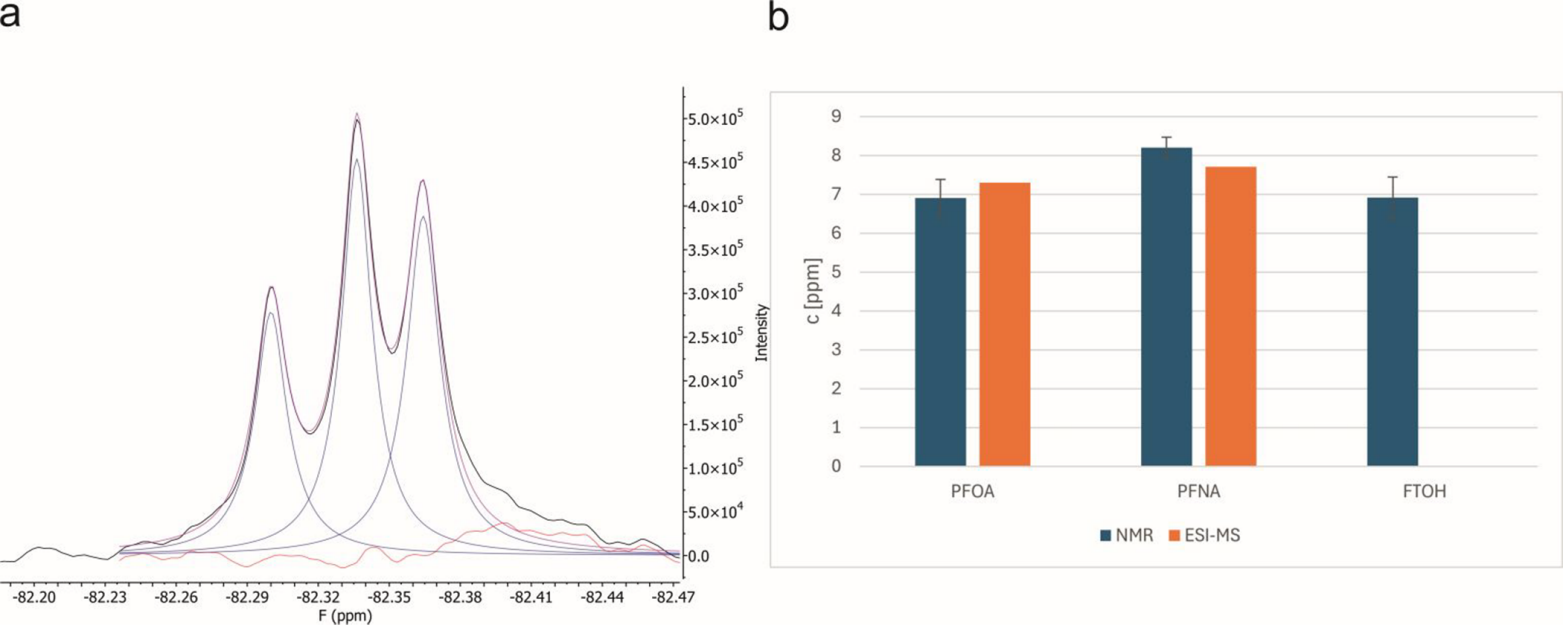
Quantification of a mix of FTOH 10:2 (δ = −82.30 ppm), PFNA (δ = −82.34 ppm), and PFOA (δ = −82.38 ppm) with a reference of PFPrA (at δ = −84.49 ppm, not shown, full spectrum in Figure S2). (a) Band-selectively homonuclear decoupled ^19^F spectrum of the CF_3_ spectral region with line fitting. (b) Comparison of the determined concentrations with ESI-MS data of the same sample mixture. FTOH 10:2 did not show up in the ESI-MS. Comprehensive experimental parameters can be found in the Supporting Information.

Using PFPrA as an internal standard was decided upon due to its close chemical shift value while still being baseline separated from the CF_3_ groups to be quantified. A potential alternative to PFPrA would be the use of a coaxial insert with hexafluoroacetone, which exhibits a similar CF_3_ chemical shift at −84.6 ppm but is considerably more hazardous. The selective excitation pulse needs to uniformly excite this specific band of the spectrum. More commonly used chemical shift referencing compounds like hexafluorobenzene cannot be employed in this setup, as the CF resonances at −164.9 ppm are too far to be excited by the same band-selective pulse.

All reported measurements were conducted at analyte concentrations that gave signal-to-noise ratios exceeding 3 within reasonable acquisition time (<10 h). This threshold served as our criterion for limit of detection (LOD).^11^ In NMR, no definitive LOD/LOQ can be given as sensitivity scales with the measurement time. With a measurement time of 9 h, our setup resulted in an LOD of 55 μg/L for the decoupled experiment and 100 μg/L of PFBA for the regular spectrum (Figure S1). Hardware improvements such as the use of a BBO H&F cryoprobe would result in a further 6-fold enhanced sensitivity according to manufacturer’s details, potentially allowing LODs below 10 μg/L.^27^ The additional use of noise canceling techniques and measurement times of 2 days should enable LODs down to 1 μg/L.^28^

Though ^19^F NMR is innately quantitative in nature, the use of band-selective decoupling influences the detected signal intensities. Diffusion and relaxation effects differentially affect signal intensities across molecules of distinct diffusion coefficients and correlation times. These factors result in a decrease in reliability of the determined concentrations. However, recently these issues have been addressed by Foster et al. in a computational effort to compensate for the effects involved, resulting in fully quantitative band-selectively decoupled ^1^H spectra.^29^ This approach has not yet been extended to ^19^F spectra but poses a further avenue to broaden the application and improve the precision of quantitative ^19^F NMR.

## 4 Conclusions

In conclusion, we present a method that allows detection and quantification of PFAS compounds at higher resolution and sensitivity than previous ^19^F NMR methods. This novel approach opens the possibility of analyzing and quantifying PFAS compounds and mixtures at concentrations hitherto inaccessible by NMR spectroscopy, especially when cryoprobes are used. The advancements presented in this work could be a step in the direction of further establishing NMR spectroscopy as a valuable method in the analysis of PFAS mixtures in complex matrices.

## Supplementary Material



## Notes

Pulse program and all other relevant experimental parameters as well as a more comprehensive list of abbreviations (PDF)

## Abbreviations

PFAS: per- and polyfluorinated alkyl substances
PFPrA: perfluoropropionic acid
PFBA: perfluorobutanoic acid
PFHxA: perfluorohexanoic acid
PFHpA: perfluoroheptanoic acid
PFOA: perfluorooctanoic acid
PFNA: perfluorononanoic acid
PFDA: perfluorodecanoic acid
PFHxS: perfluorohexanesulfonic acid
FTOH: fluorotelomeric alcohol
BASH: band-selective homonuclear
LOD: limit of detection
LOQ: limit of quantification
