# Supplementary material for: Enhancing both the Intensities and Resolution of 19F NMR Spectra of PFAS through Band-Selective Homonuclear Decoupling
Source: Anal Chem. 2026 Jan 27;98(5):3821–6. doi: 10.1021/acs.analchem.5c05889 (PMC12903055; doi:10.1021/acs.analchem.5c05889)
Supplement: Supplementary file 1 [file ac5c05889_si_001.pdf]

Supporting information:

Enhancing both the Intensities and Resolution of ^{19}F NMR Spectra of PFAS through Band-selective Homonuclear Decoupling

Markus Rotzinger[a], Viktoria Müller[b,c], Armin Macher[a], Jörg Feldmann[b] and Klaus Zangger*[a]

[a] Institute of Chemistry/Organic and Bioorganic Chemistry, University of Graz, Heinrichstraße 28, 8010 Graz, Austria

[b] Institute of Chemistry/Analytical Chemistry, University of Graz, Universitätsplatz 1, 8010 Graz, Austria

[c] The James Hutton Institute, Craigiebuckler, Aberdeen AB15 8QH, United Kingdom

Table of Contents:

NMR Measurements....3
Determination of the limit of detection: 5
Resolution limit testing....7
LC-ESI-MS/MS Measurements....8
Chemicals....8
Instrumentation....9
Quality control....9
Pulseprogram in Bruker notation: 14

NMR Measurements

Stock solutions of the solid PFAS samples were prepared in methanol. Solutions of perfluorocarboxylic acids were neutralized using an equimolar amount of sodium hydroxide to prevent esterification. The mixed samples were inserted into a 5 mm sample tube from the stock solutions. A solution of chromium (III) acetylacetonate in methanol-d_4 up to a final chromium (III) acetylacetonate concentration of 4 mg/mL and a volume content of methanol-d_4 of 150 µL was added to enhance the T_1 relaxation times and thus allow for short relaxation delays between scans. The sample was filled with methanol-d_4 to a total volume of 550 µL. Methanol-d_4 was purchased from eurisotop.

All spectra were acquired on a 500 MHz Bruker Avance Neo spectrometer equipped with a 5 mm SEF ^{19}F probe at 298 K. ^{19}F hard pulses were calibrated iteratively from a 360° pulse to 5 µs. Soft pulses with a flip angle of 90° were calculated from the determined hard pulses.

The spectra for the mixture of PFAS compounds at 1.5 ppm (figure 3) were acquired using 16k transients with a FID size of 8K complex time-domain points. The relaxation delay was set to 1 s for spectra intended for qualitative interpretation. The offset was set to -82.3 ppm for the mixtures of compounds with a band-selective pulse excitation bandwidth of 2500 Hz. Quantitative measurements (figure 4) proceeded using an identical setup, while acquiring 1k transients and extending the relaxation time to 10 seconds to enable sufficient relaxation between individual scans. Detailed parameters for each spectrum are given in Table S1. Sample concentrations were adjusted to around 10 mg/L while the PFPrA concentration used as internal standard was 1 mg/L. The resulting concentrations were derived from a comparison of the fitted integral of the individual peaks with the internal standard peak and adjusting for different molecular mass. Measurements were recorded as triplicates under identical conditions in direct succession. $CFCl_3$ was added as an internal standard at a concentration of 2 mg/L.

The samples for the ESI-MS measurements were prepared by diluting a mixture of PFOA:PFNA:PFPrA at a ratio of 10:10:1 in two steps by a factor of 1000 to afford a sample concentration 10 ppb for the long chain PFCAs.

Table S1: Experimental parameters used for the measurements shown in the main text figures. NS = number of transients, FID = FID size, D1 = interscan delay, O1 = offset, AQ = acquisition time, SW = sweep width, L0 = loop count, t chunk = chunking time, BW = bandwidth, Expt = total time for the measurement according to Topspin

	**Figure 1**		**Figure 3**				**Figure 4 Mix**		**individual**		**Figure 5**
**concentration**	1000 mg/L	1000 mg/L	1.5 mg/L	1.5 mg/L	100 mg/L	100 mg/L	100 mg/L	1.5 mg/L	330 mg/L	330 mg/L	10 mg/L
**type**	^{19}F	decoupled	^{19}F	decoupled	^{19}F	decoupled	^{19}F	decoupled	^{19}F	decoupled	decoupled
**NS**	256	256	16384	16384	4096	128	4096	16384	32	32	1024
**FID**	262144	8192	262144	8192	262144	8192	262144	8192	32768	8192	8192
**D1 [s]**	2.00	2.00	0.50	0.50	0.50	1.50	0.50	0.50	2	1	10.00
**O1 [ppm]**	-100.00	-82.33	-100.00	-82.33	-100.00	-82.33	–100,00	-82.33	-82.33	-82.33	-83.50
**AQ [s]**	2.78	0.87	2.78	0.87	2.78	0.87	2.78	0.87	3.47	0.87	0.87
**SW [ppm]**	100.00	10.00	100.00	10.00	100.00	10.00	100.00	10.00	10.00	10.00	10.00
**L0**		40.00		40.00		40.00		40.00		35.00	40.00
**t chunk [ms]**		21.70		21.70		21.70		21.70		24,48	21.70
**BW [Hz]**		540.00		540.00		540.00		540.00		2500	2500
**Expt**	20 min	13 min	15 h 3 min	7 h 35 min	3 h 46 min	6 min	3 h 46 min	7 h 35 min	3 min	2 min	3h 10 min

Determination of the limit of detection:

A 100 µg/L sample of perfluorobutyric acid (PFBA) with 2 mg/mL $Cr[Acac]_3$ was prepared and measured using optimized parameters (16k transients, 8k FID size, interscan delay 1 s, chunking time 24.8 ms, loop count 35, acquisition time 0.87 s, CNST12 0.85, CNST13 1.3, total experiment time 9 h 27 min). Subsequent comparison with a coupled ^{19}F spectrum (16 k transients, 8 k FID size acquisition time 0.87 s, interscan delay 1 s, total experiment time 8h 38 min) showed a significant increase in signal to noise ratio as well as spectral resolution due to decoupling. The signal to noise ratio was calculated using the function integrated in the Topspin software termed 'sino'. Signal to noise ratio was calculated to 5.4 for the decoupled experiment and 2.7 for the regular spectrum. Extrapolation of the results to the concentration which gives a signal to noise ratio of 3.0 with identical parameters gives 55 µg/L as a limit of detection.

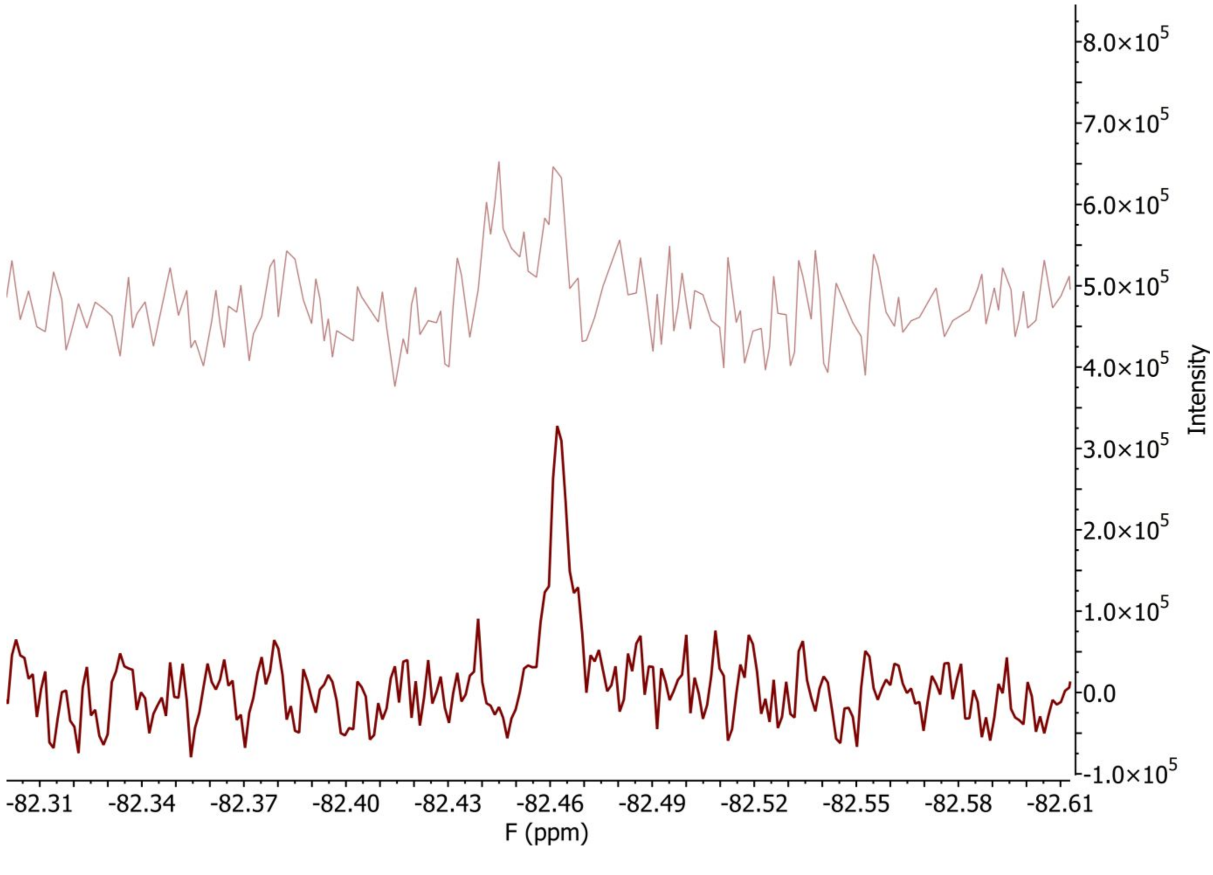

Figure S1. Stacked spectra of decoupled and coupled PFBA sample. The decoupled spectrum is depicted in maroon and the regular spectrum is shown vertically shifted and in lighter hue.

Table S2: Experimental parameters of determination of LOD, NS = number of transients, FID = FID size, D1 = interscan delay, O1 = offset, AQ = acquisition time, SW = sweep width, L0 = loop count, t chunk = chunking time, BW = bandwidth, Expt = total time for the measurement according to Topspin

	Figure S1	
concentration	100 µg/L	100 µg/L
type	^{19}F	decoupled
NS	16k	16k
FID	8k	8k
D1 [s]	1	1.00
O1 [ppm]	-82.45	-82.45
AQ [s]	0.87	0.87
SW [ppm]	10.00	10.00
L0		35.00
t chunk [ms]		24.48
BW [Hz]		2500.00
Expt	8 h 38 min	9 h 27 min

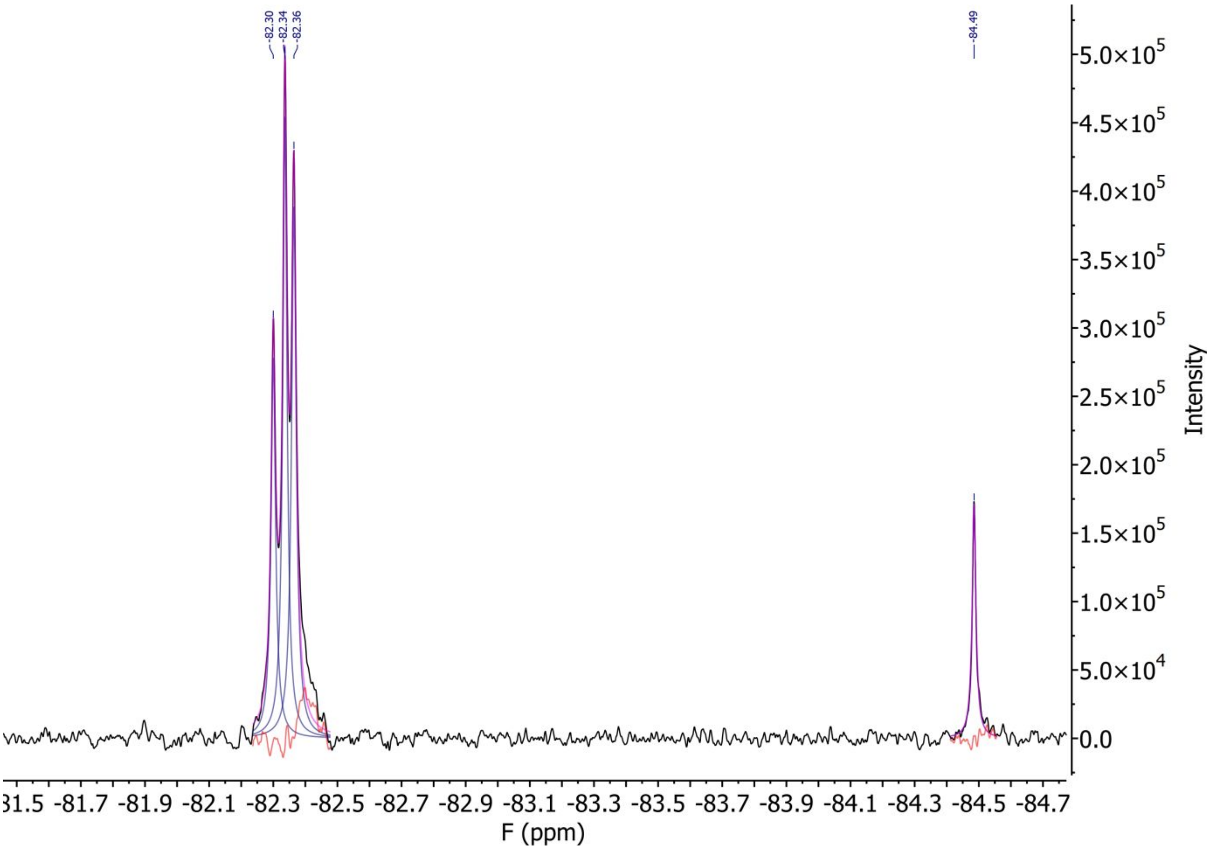

Figure S2. Full spectrum used for quantification including the reference signal of PFPrA at -84.5 ppm.

Resolution limit testing

To evaluate performance in complex sample matrices, the HPLC recovery standard PFAC30PAR (constituents listed in chemicals for LC-ESI-MS/MS-measurements and contained in Table S4) was analysed using an experimental setup comparing a band-selective decoupled spectrum with a conventional ^{19}F NMR spectrum. Analogous parameters as in previous experiments (Table S2) were employed regarding the calibration of the decoupling as well as the acquisition to ensure comparable results. The sample was prepared from 400 µL of the standard, with 2 mg/mL Cr[acac]$_3$ and the total addition of 150 µL methanol-d$_4$.

Although the decoupled experiment provided a substantial improvement in spectral resolution, signal overlap in the −82.3 to −82.5 ppm region remained too pronounced to achieve baseline separation. As a result, without access to substantially higher magnetic field strengths, mixtures of this complexity cannot be sufficiently resolved to enable unambiguous assignment of individual resonances.

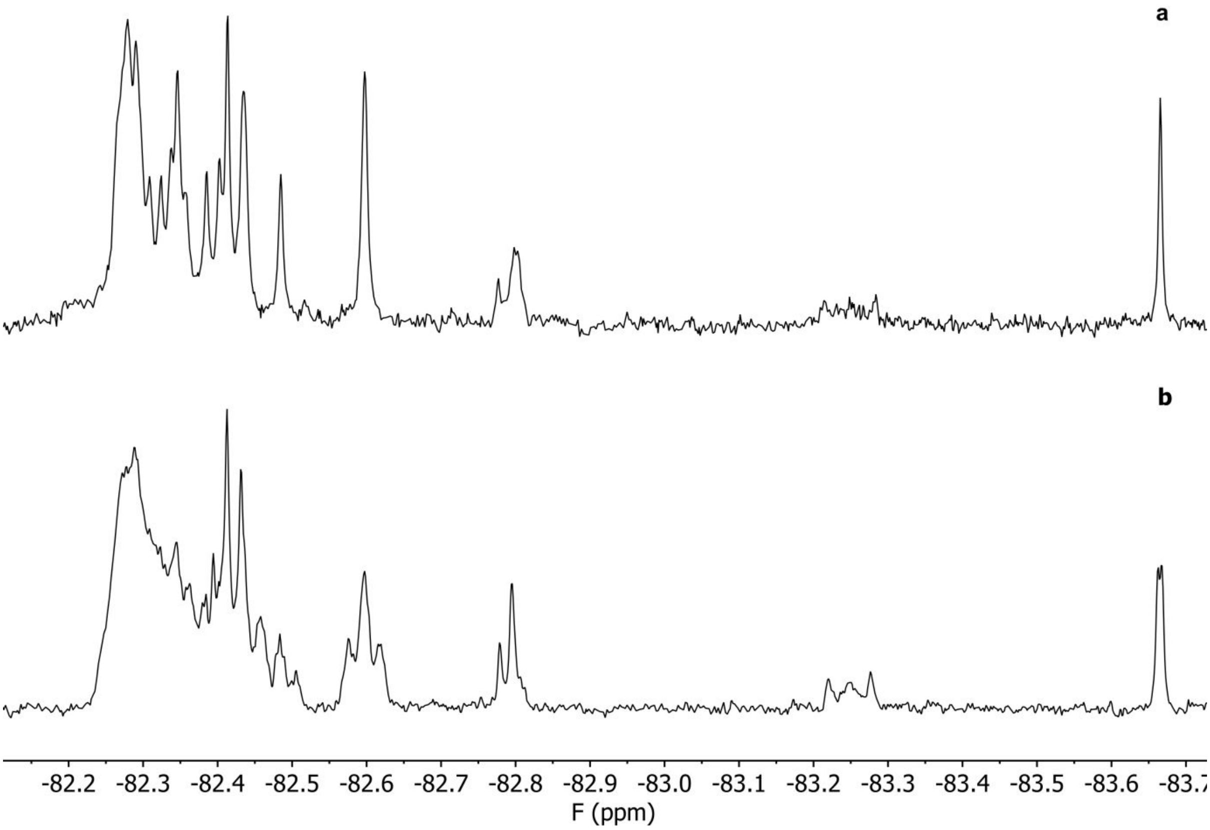

Figure S3. Comparison of a) the band-selectively decoupled spectrum and b) the regular ^{19}F spectrum of the PFAC30PAR mixture

Table S3: Acquisition parameters for resolution limit testing in Figure S3., NS = number of transients, FID = FID size, D1 = interscan delay, O1 = offset, AQ = acquisition time, SW = sweep width, L0 = loop count, t chunk = chunking time, BW = bandwidth, Expt = total time for the measurement according to Topspin.

**Figure S3**	**a**	**b**
**concentration**	1 mg/L	1 mg/L
**type**	decoupled	^{19}F
**NS**	16384	16384
**FID**	8k	8k
**D1 [s]**	1	1.00
**O1 [ppm]**	-84.5	-83.0
**AQ [s]**	0.87	0.87
**SW [ppm]**	10.00	10.00
**L0**	35.00	
**t chunk [ms]**	24.48	
**BW [Hz]**	5000	
**Expt**	9 h 27 min	8 h 38 min

LC-ESI-MS/MS Measurements

Chemicals

PFAS standards include a native PFAS mix (PFAC30PAR), which contains perfluoro carboxylic acids (PFCA) ranging from C4 – C14, perfluorosulfonic acids (PFSA) ranging from C4 – C10, C4, C6 and C8 perfluoroalkanesulfonamides, methyl perfluoro octanesulfonamidoethanol, and ethyl perfluorooctanesulfonamidoethanol, GenX, Nadona, 9Cl-PF3ONS, and 11Cl-PF3OUdS. MPFAC-HIF-ES which contains ^{13}C labelled C4 – C12, C14 PFCA and C4, C6, C8 PFSA, C4, C6, C8 perfluorosulfonates (FTS), GenX, methyl perfluoro octanesulfonamide, ethyl perfluorooctanesulfonamide, methyl perfluoro octanesulfonamidoethanol, and ethyl perfluorooctanesulfonamidoethanol. Were purchased from Wellington Laboratories (Canada). Ultrapure water was provided from Millipore Ultrapure

Water System (18 MΩ cm, Merck). Ammonium acetate (CH_3COONH_4) LC/MS grade was purchased from Fischer Chemicals (Austria). Methanol (MetOH) and acetonitrile (ACN), both LC/MS grade, were purchased from Bartelt (Austria).

Instrumentation

Targeted analysis with LC-ESI-MS/MS

An Agilent 1260 infinity II HPLC (Agilent Technologies, Germany) combined with a BrownLee SPP C18 column (2.7 µm, 3 x 100 mm, PerkinElmer, UK) and a BrownLee SPP guard column (2.7 µm, 3 x 5 mm, PerkinElmer, UK) were used for the separation of the analytes by LC. A gradient was used for the separation of target PFAS, consisting of 5 mmol L^{-1} CH_3COONH_4. The LC system was coupled to an Agilent Ultivo LC/TQ (Agilent Technologies, Germany). The MS was used in negative multiple response monitoring (MRM) mode. Two transitions were monitored (quantifier and qualifier ions) for the analytes except PFBA, PFPA, PFdDA, PFOSA, and the ultrashort chain PFAS where only one transition was monitored. Transitions and LC methods are listed in Table SX in SI. Due to the large number of transitions, the transitions were monitored only around their retention times.

Quality control

Samples were spiked with 10 µL of 16 – 40 µg L^{-1} HIF-ES mix and were analysed with the LC-MS/MS. In case of observing PFAS in the blanks, the average area of the method blanks was subtracted from the samples. A calibration curve with seven points, ranging from 0.05 ng g^{-1} to 50 ng g^{-1} in methanol was measured during the run. Quantification was performed using the analyte:isotopically labelled internal standard ratio. The instrumental limit of quantification and detection (LOQ and LOD) were calculated as ten times and three times the error of the linear regression divided by the slope, respectively.

Table S4. List of analytes and their acronyms

**PFAS carboxylic acids**	**PFCAs**	
Perfluorobutanoic acid	PFBA	C_4
Perfluoropentanoic acid	PFPA	C_5
Perfluorohexanoic acid	PFHxA	C_6
Perfluoroheptanoic acid	PFHpA	C_7
Perfluorooctanoic acid	PFOA	C_8
Perfluorononanoic acid	PFNA	C_9
Perfluorodecanoic acid	PFDA	C_{10}
Perfluoroundecanoic acid	PFuDA	C_{11}
Perfluorododecanoic acid	PFdDA	C_{12}
Perfluorotridecanoic acid	PFtrDA	C_{13}
Perfluorotetradecanoic acid	PFteDA	C_{14}
Perfluorohexadecanoic acid	PFhxDA	C_{16}
Perfluorooctadecanoic acid	PFoDA	C_{18}
**PFAS sulfonic acids**	**PFSAs**	
Perfluoro-1- butanesulfonic acid	PFBS	C_4
Perfluoro-1-pentanesulfonic acid	PFPS	C_5
Perfluoro-1-hexanesulfonic acid	PFHxS	C_6
Perfluoro-1-heptanesulfonic acid	PFHpS	C_7
Perfluoro-1-octanesulfonic acid	PFOS	C_8
Perfluoro-1-nonanesulfonic acid	PFNS	C_9
Perfluoro-1-decanesulfonic acid	PFDS	C_{10}
Perfluoro-1-dodecanesulfonic acid	PFDoS	C_{12}
**PFAS sulfonamides and sulfonamidoacetic acids**		
Perfluoro-1-octanesulfonamide substances	PFOSA	
Perfluorooctanesulfonamidoacetate	PFOSAA	
N-ethyl perfluorooctyl sulfonamide	*N*-EtFOSA	
2-(*N*-Methylperfluoro-1-octanesulfonamido)-ethanol	*N*-MeFOSE	
2-(*N*-ethylperfluoro-1-octanesulfonamido)-ethanol	*N*-EtFOSE	
**Others**		
4,8-Dioxa-3H-perfluorononanoic acid	ADONA	
Hexafluoropropylene oxide dimer acid	GenX	
Sodium 1H, 1H, 2H, 2H-perfluorohexane sulfonate	4:2 FTS	
Sodium 1H, 1H, 2H, 2H-perfluorooctane sulfonate	6:2 FTS	
Sodium 1H, 1H, 2H, 2H-perfluorodecane sulfonate	8:2 FTS	
Na 1H, 1H, 2H, 2H-perfluorododecane sulfonate	10:2 FTS	

Table S5. List of monitored transitions and MS parameters.

Compound Name	Precursor ion (m/z)	Product ion (m/z)	Ret Time (min)	Fragmentor	Collision Energy (V)	Polarity
4_2 FTS	327	307	6.3	94	20	Negative
4_2 FTS	327	81	6.3	94	36	Negative
6_2 FTS	427	407	7.7	128	24	Negative
6_2 FTS	427	81	7.7	128	56	Negative
8_2FTS	527	507	9.7	134	28	Negative
8_2FTS	527	81	9.7	134	68	Negative
9 Cl-PF3ONS	531	351	11.4	132	28	Negative
9 Cl-PF3ONS	531	83	11.4	132	32	Negative
10_2FTS	627	607	13.7	168	36	Negative
10_2FTS	627	81	13.7	168	68	Negative
11 Cl-PF3OUdS	631	451	14	162	32	Negative
11 Cl-PF3OUdS	631	83	14	162	36	Negative
d N EtFOSA	531	169	13.2	132	32	Negative
d N MetFOSA	515	169	12	134	32	Negative
d3 N-MetFOSAA	573	419	10.7	94	20	Negative
d5 N-EtFOSAA	589	419	11.4	94	20	Negative
d7 N MetFOSE	623	59	19.9	96	52	Negative
d9 N EtFOSE	639	59	20.3	96	68	Negative
FBSA	298	78	7.7	94	32	Negative
FHxSA	398	78	14.1	94	60	Negative
FOSAA	556	498	11.5	136	32	Negative
FOSAA	556	419	11.5	136	28	Negative

GenX	285	185	6.6	64	16	Negativ e
GenX	285	169	6.6	64	4	Negativ e
M2 4_2 FTS	329	309	6.3	94	20	Negativ e
M2 6_2 FTS	429	409	7.7	130	24	Negativ e
M2 8_2 FTS	529	509	9.7	162	28	Negativ e
M2PFOA	415	370	7.9	70	4	Negativ e
M2PFteDA	715	670	15.5	100	8	Negativ e
M3HFPO-DA	287	169	6.6	50	4	Negativ e
M3PFBA	216	172	3.7	50	4	Negativ e
M3PFBS	302	80	6.5	130	48	Negativ e
M3PFHxS	402	80	8.2	132	68	Negativ e
M4PFHpA	367	322	7.1	66	4	Negativ e
M5PFHxA	318	273	6.5	64	4	Negativ e
M5PFPEA	268	223	6	50	4	Negativ e
M6PFDA	519	474	11	130	16	Negativ e

Table S5. Continued

**Compound Name**	**Precursor ion (m/z)**	**Product ion (m/z)**	**Ret Time (min)**	**Fragmentor**	**Collision Energy (V)**	**Polarity**
M8PFOA	421	376	7.9	76	8	Negative
M8PFOS	507	80	10.2	164	92	Negative
M9PFNA	472	427	9	70	8	Negative
MPFBA	217	172	3.7	50	4	Negative
MPFDA	515	470	11	168	104	Negative
MPFdDA	615	570	12.8	96	8	Negative
MPFOS	503	80	11.8	162	92	Negative
NaDONA	377	251	7.4	68	8	Negative
NaDONA	377	85	7.4	68	44	Negative
N-EtFOSA	526	219	20.6	132	28	Negative

N-EtFOSA	526	169	20.6	132	32	Negative
N-EtFOSAA	584	483	11.4	94	12	Negative
N-EtFOSAA	584	419	11.4	94	20	Negative
N-EtFOSE-M	630	59	20.5	98	68	Negative
N-MetFOSAA	570	483	10.7	94	16	Negative
N-MetFOSAA	570	419	10.7	94	20	Negative
N-MetFOSE-M	616	59	20.1	98	76	Negative
PFBA	213	169	3.7	40	4	Negative
PFBA	213	69	3.7	40	44	Negative
PFBS	299	99	6.5	130	36	Negative
PFBS	299	80	6.5	130	52	Negative
PFDA	513	469	11	74	8	Negative
PFDA	513	219	11	74	16	Negative
PFdDA	613	529	12.8	96	8	Negative
PFdDA	613	219	12.8	96	28	Negative
PFdDA	613	169	12.8	96	28	Negative
PFdDS	699	99	16	166	116	Negative
PFdDS	699	80	16	166	116	Negative
PFDS	599	99	13.1	178	88	Negative
PFDS	599	80	13.1	178	108	Negative
PFHpA	363	319	7.1	66	4	Negative
PFHpA	363	169	7.1	66	16	Negative
PFHpS	449	99	9.3	164	60	Negative
PFHpS	449	80	9.3	164	72	Negative
PFHxA	313	269	6.5	64	4	Negative
PFHxA	313	119	6.5	64	4	Negative
PFhxDA	813	769	16.5	130	20	Negative
PFhxDA	813	169	16.5	130	32	Negative
PFHxS	399	99	8.2	136	52	Negative
PFHxS	399	80	8.2	136	72	Negative
PFNA	463	419	9	72	8	Negative
PFNA	463	169	9	72	20	Negative

Table S5. Continued

**Compound Name**	**Precursor ion (m/z)**	**Product ion (m/z)**	**Ret Time (min)**	**Fragmentor**	**Collision Energy (V)**	**Polarity**
PFNS	549	99	11.7	174	56	Negative
PFNS	549	80	11.7	174	108	Negative
PFOA	413	369	7.9	68	4	Negative
PFOA	413	169	7.9	68	16	Negative
PFoDA	913	869	17.5	130	104	Negative
PFoDA	913	169	17.5	130	104	Negative
PFOS	499	99	10.5	162	68	Negative
PFOS	499	80	10.5	162	84	Negative
PFOSA	498	78	18	130	72	Negative
PFOSA	498	48	18	130	72	Negative

PFPEA	263	219	6	50	50	Negative
PFPEA	263	169	6	50	50	Negative
PFPS	349	99	7.2	132	40	Negative
PFPS	349	80	7.2	132	60	Negative
PFteDA	713	669	15.4	100	12	Negative
PFteDA	713	169	15.4	100	32	Negative
PFtrDA	663	619	14	98	8	Negative
PFtrDA	663	169	14	98	32	Negative
PFuDA	563	519	11.5	78	8	Negative
PFuDA	563	119	11.5	78	48	Negative

Table S6. LC method

**Time (min)**	**A (%)**	**B (%)**
0	97	3
1	97	3
2	70	30
14	40	60
17.5	0	100
23.5	0	100
23.6	97	3
28	97	3

Pulseprogram in Bruker notation:

```
;zgadc2
;avance-version (06/01/20)
;1D sequence with explicit programming of acquisition
;
;$CLASS=HighRes
;$DIM=1D
;$TYPE=
;$SUBTYPE=
;$COMMENT=


#include <Avance.incl>
#include <De.incl>
#include <Grad.incl>
```

```
#include <Delay.incl>

;dwellmode explicit

;define list<shape> shl1=<Eburp2.1000>
;define list<shape> shl2=<Gaus_180r.1000>

"d11=3u"
"d12=6.5u"
"d20=10u"
"d2=aq/l0"
"d3=d2/2"
"l1=l0-1"
"p2=2*p1"


#   ifdef CALC_SP
"p48=(bwfac36/(cnst11*cnst13))*1000000"
"spw36=plw1/(((p48*90.0)/(p1*totrot36))*((p48*90.0)/(p1*totrot36))*(integfac36*integfac36))"
"spoal36=0.5"

"spnam1='Eburp2.1000'"
"p12=(bwfac1/(cnst11*cnst12))*1000000"
"spw1=plw1/((p12*90.0)/(p1*totrot1))*((p12*90.0)/(p1*totrot1))*(integfac1*integfac1)"
"spoal1=1"

"spoffs36=0"
"spoffs1=0"
#   endif /*CALC_SP*/


1 ze
2 3m
  4u BLKGRAD
  d1 rpp2
  50u UNBLKGRAD

  d12 pl0:f1
  d12 gron1
  (p12:sp1 ph1)
  d12 groff
  d12 pl1:f1



  ACQ_START(ph30,ph31)
  0.1u START_NEXT_SCAN

   0.05u DWELL_RELEASE
   0.1u REC_UNBLK
   d3:r
   0.1u REC_BLK
   0.05u DWELL_HOLD

   p16:gp2
```

```
   d16 pl1:f1
   p2 ph2
   p16:gp2
   d16

   p16:gp3
   d16 pl0:f1
   10u
   d11 gron1
   (p48:sp36 ph3)
   d11 groff
   10u
   p16:gp3
   d16

3  0.05u DWELL_RELEASE
   0.1u REC_UNBLK
   d2:r
   0.1u REC_BLK
   0.05u DWELL_HOLD

   p16:gp2
   d16 pl1:f1
   p2 ph2
   p16:gp2
   d16

   p16:gp3
   d16 pl0:f1
   10u
   d11 gron1
   (p48:sp36 ph3)
   d11 groff
   10u
   p16:gp3
   d16

lo to 3 times l1

  0.05u DWELL_RELEASE
   0.1u REC_UNBLK
   d3
   25m
   0.1u REC_BLK
   0.05u DWELL_HOLD



rcyc=2


wr #0

exit
```

```
ph1 =0 2 2 0 1 3 3 1
ph2 = 0 2
ph3 = 2 0
ph30=0
ph31=0 2 2 0 1 3 3 1


;pl1 : f1 channel - power level for pulse (default)
;p1 : f1 channel -  high power pulse
;d1 : relaxation delay; 1-5 * T1
;cnst11: bandwidth for selective proton pulse (e.g. 90 Hz)
;cnst12: scaling factor for selective 90 deg. pulse (e.g. 0.9)
;cnst13: scaling factor for selective 180 deg pulse (e.g. 1.1)
;NS: 1 * n, total number of scans: NS * TD0
;l20:


;$Id: zgadc,v 1.12 2009/07/02 16:40:47 ber Exp $
```
